# Prevalence and knowledge of modifiable cardiovascular diseases risk factors among vulnerable population in Central Tanzania

**DOI:** 10.1186/s12872-023-03408-3

**Published:** 2023-07-26

**Authors:** Nyasiro Sophia Gibore, Mariam John Munyogwa, Secilia Kapalata Ng’weshemi, Ainory Peter Gesase

**Affiliations:** 1 Department of Public Health and Community Nursing, The University of Dodoma, P.O. Box 395, Dodoma, Tanzania; 2 Department of Community Medicine, The University of Dodoma, P.O. Box 395, Dodoma, Tanzania; 3 Department of Anatomy and Histology, The University of Dodoma, P.O. Box 395, Dodoma, Tanzania

**Keywords:** Cardiovascular diseases risk factors, Modifiable risk factors, Biological risk factors, Behavioral risk factors, Knowledge of risk factors, Obesity

## Abstract

**Background:**

Cardiovascular diseases (CVDs) are the global public health problem which has been associated with increasing prevalence of modifiable CVDs risk factors. This study aimed to describe the prevalence and knowledge of modifiable CVDs risk factors among vulnerable population of Central Tanzania.

**Methods:**

A community-based cross-sectional study design was employed. A total of 749 participants were interviewed. The socio-demographic information and modifiable CVDs risk factors (behavioral and biological) were measured using a modified World Health Organization (WHO) STEPwise approach for chronic disease risk factor surveillance. Knowledge of modifiable CVDs risk factors was measured by comprehensive heart disease knowledge questionnaire. Descriptive statistics were used to describe the knowledge and prevalence of modifiable CVDs risk factors. Logistic regression analysis was used to determine the factors associated with adequate knowledge of CVDs risk factors.

**Results:**

The prevalence of béhavioral risk factors were; current smokers and alcohol consumers were 4.4% and 18.0% respectively, use of raw salt was 43.7%, consumption of fruit/vegetables < 5 days per week was 56.9%. The prevalence of Biological CVDs risk factors was as follows: Overall, 63.5% (33.3% overweight and 29.9% obese) were overweight or obese, 4.5% were diabetic and 43.4% were hypertensive. Only 35.4% of participants had adequate knowledge of CVDs risk factors. Being a male (AOR = 1.44, 95%CI = 1.01–2.06, p < .05), having primary education (AOR = 6.43, 95%CI = 2.39–17.36, p < .0001), being employed (AOR = 1.59, 95%CI = 1.00-2.52, p < .05), ever checked blood pressure (AOR = 0.59, 95%CI = 0.42–0.84, p < .001), family history of hypertension (AOR = 0.38, 95%CI = 0.25–0.57, p < .0001) determined adequate knowledge of CVDs risk factors.

**Conclusions:**

This study has revealed a high prevalence of modifiable CVDs risk factors and low knowledge of CVDs risk factors. Community health promotion interventions to increase population knowledge of CVDs risk factors are recommended for the efficacious reduction of CVDs in the country.

## Background

Cardiovascular diseases (CVDs) are a group of disorders of the heart and blood vessels that include coronary artery disease, stroke, heart failure, hypertensive heart disease, rheumatic heart disease, cardiomyopathy, valvular heart disease, and peripheral artery disease [1]. These diseases are common in the general population, mainly among adults population aged < 70 years, with stroke and coronary heart diseases being the main contributors [1, 2]. CVDs are the global public health problem, accounting for 32% of all deaths in 2019, with an alarming 80% of these deaths occurring in low- and middle-income countries [3]. It has been projected that, if appropriate measures are not undertaken, by the year 2030, CVDs will cause more than 23.6 million deaths [4].

In sub-Saharan Africa most of the population risk of CVDs is fueled by modifiable risk factors, such as smoking, hypertension, diabetes, overweight or obesity, unhealthy diet, being physical inactive, excessive alcohol consumption, raised blood lipids and psychosocial factors [5]. In Tanzania, increased urbanization, lifestyle changes, lack of awareness and rural to urban movement have been found to increase CVDs risk factors [6]. The urban population is more vulnerable due to sedentary lifestyles, higher incidence of overweight, obesity, and elevated blood cholesterol levels than rural population [7]. Studies have shown that alleviating exposure to these risk factors would improve global life expectancy by almost 5 years [8, 9].

In order to prevent or delaying the risk of developing CVDs and improve in cardiovascular health, a public health literacy of CVDs risk factors is required [10]. Studies have shown that health literacy of CVDs risk factors can lead to success in their prevention and control through influencing individual practices towards healthy lifestyle modification, impacting the efficacy of their management and decrease risk of disease complications [11–13]. It is evident that individuals with inadequate health knowledge have been associated with decreased use of health care services and less likely to engage in health promotion behaviors resulting in overall poor health outcomes [12, 13].

Whilst the world health organization is promoting knowledge of behavioral CVDs risk factors [1]. a systemic review of studies conducted in sub-Saharan Africa (SSA) have reported low levels of knowledge on CVDs risk factors [14]. In Tanzania a study conducted in rural population of Morogoro Region found only quarter of participants (25.4%) had good knowledge on CVDs risk factors [10]. Another hospital-based study conducted among companions of outpatients attending a tertiary-level cardiovascular hospital in Dar es Salaam Tanzania found that about 80.0% of study participants had good knowledge of CVDs risks factors [15]. Despite the increasing in CVDs deaths and their related risk factors in urban population of Tanzania [6], there is dearth of information regarding public knowledge of CVDs risk factors among urban population. Information on community knowledge regarding CVDs risk factors will serve as the basis for community mobilization for health education intervention strategies for preventing exposure to CVDs risk factors and resulting in reduction of the growing burden of CVDs. This study assessed prevalence and knowledge of modifiable cardiovascular diseases risk factors and factors associated among the vulnerable population in central Tanzania.

## Methods

### Study design and setting

A baseline survey was conducted between March to May, 2021 as part of a community-based cluster randomized controlled trial study on the effectiveness of a community-based lifestyle intervention in reducing the threats of developing CVDs risk factors among vulnerable population in Dodoma City as described in [16]. The current study analysed the baseline data before the intervention. The survey was conducted in four randomly selected wards (Nkhuhungu, Chang’ombe, Tambukareli and Ipagala). Based to the population projection of the 2017, Dodoma city was projected to have a population of 522,428 in 2022 with the average annual population growth rate of 2.4% [17]. The city is inhabited by several ethnic groups, this is due to rapid urbanization which has resulted in a large movement of people from all over the country to Dodoma city. However, the predominant indigenous ethnic groups are Gogo, Rangi and Sandawe whose main economic activities include businesses, seasonal agriculture that depends on unimodal rainfall and agro-pastoral activities. The main staple foods include stiff porridge from maize, sorghum or millet served with dried green vegetables and milk in some families.

### Study participants

The study participants were male and female adults aged 25–88 years, residents of the selected ward for a period of more than four months, who consented verbally or by writing to participate in the study. Pregnant women, seriously sick and mentally ill individuals were excluded in the study.

### Sample size estimation and sampling technique

The sample size was estimated to provide a 90% power to detect a proportional change in body BMI of 20% between the intervention and control groups at an alpha level of 5% and 95% confidence interval, an expected drop-out of 20%, an intercluster correlation of 0.03, and a standard deviation of 0.84, with the ratio of intervention to control of 1:1. The resulting minimum sample size for the study was 400. The study assigned 400 participants in an intervention group and another 400 participants in a control group. Therefore, the total sample size for the study was 800 participants. Simple random sampling using lottery method was used to select four wards. From each ward a systemic sample of 200 households were selected from a list of all households with eligible participants that was identified by the help of respective street leaders. The lottery method was used to select one respondent for households with more than one eligible respondent. If the selected individual in the household was unable to be interviewed, the next household on the list was chosen.

### Data collection tools and procedure

Trained research assistants conducted face-to-face interviews using an electronic structured questionnaire which was developed in the open-source toolkit (KoBoToolbox) for data collection and it was accessed through mobile phone. A team of research assistants consisted of the following professionals; registered nurses, nutritionist, public health specialist and laboratory technicians collected the data. They were trained on how to collect data using a mobile phone, as well as on the study instruments and the overall data collection process. Before beginning data collecting, two weeks of training were completed. The questionnaire was adapted from WHO-steps survey [18] and comprehensive heart disease knowledge questionnaire [19], pretested and modified to meet the study environment. The following information was collected during the interview: - Socio-demographic information, knowledge of modifiable CVDs risk factors, behavioral characteristics, physical measurements and medical history. STEPS Instrument from WHO STEPS Surveillance manual [18] collected information on socio-demographic information, behavioral characteristics, physical measurements and medical history while the comprehensive heart disease knowledge questionnaire [19] was used to measure the knowledge of modifiable CVDs risk factors. To ensure content-related validity, the questionnaire was translated into Kiswahili language by two professional experts in the field of the study who were fluent in both Kiswahili and English. It was further critically evaluated by two other experts in the field so as to establish face validity.

### Assessment of knowledge on modifiable CVDs risk factors

Knowledge of modifiable CVDs risk factors was assessed using 11 multiple choice questions related to CVDs risk behaviors and prevention. Knowledge scores were assigned as 1 (one) for each correct answer and 0 (zero) for wrong answer. A percentage score for each participant was calculated by dividing the sum of correct answers to the total number of questions and converted to percentage by multiplying with 100. A score of < 50% was classified as “inadequate knowledge” and a score of ≥ 50% as “adequate knowledge”.

### Medical history records and behavioural risk factors Assessment

Participants were asked if they had ever had their blood pressure or blood sugar checked and if they had ever been diagnosed with hypertension or diabetes mellitus. Behavioral risk factors included alcohol consumption, smoking and dietary habits as well as use of raw table salt. Alcohol consumption status was assessed currently, past or never drunk. Smoking habits were assessed currently, past or never smoke. Dietary assessment was based on frequency of consumption of fruits and vegetables in days per week and frequency of use of raw table salt was assessed as never, sometimes or always.

### Physical measurements of biological risk factors

Anthropometric measurements taken were weight (kg) and height (cm). Weight was measured to the nearest 0.1 kg using a digital scale (SECA®) and height was taken to the nearest 0.1 cm using a portable stadiometer (SECA®) placed on flat ground [20]. Height measurements were taken with heels, buttocks and upper back in perpendicular with the stadiometer [20]. Before taking measurements, participants were coached to stand in the device while having light clothing and with no shoes. Calibration of equipment was performed on a daily basis in accordance with the manufacturer’s instructions. Body mass index (BMI) was calculated using measured weight (in kg) divided by height (in meters-squared). A person was considered to be underweight if BMI < 18.5 kg/m2, normal: BMI 18.5–24.9 kg/m2, overweight: BMI 25.0– 29.9 kg/m2 and obese: BMI ≥ 30.0 kg/m2 [20]. Blood pressure was measured for three readings using a digital blood pressure (OMRON®) machine, each separated by 5 min of rest in between and with arms elevated at heart level. The average of the second and the third reading was used. Participant was considered hypertensive if had a diastolic blood pressure of ≥ 90 mmHg, and/or systolic blood pressure of ≥ 140 mmHg and/or currently taking blood pressure lowering agent [18, 21]. Blood glucose measurement was taken with a digital glucose meter (GlucoPlus) machine following the procedure explained in the user guide manual [22]. Diabetes was diagnosed using a random blood glucose (RBG) ≥ 11.1 mmol/L and/or fasting blood glucose (FBG) ≥ 7 mmol/L or use of glucose-lowering agents [18, 23].

### Data analysis

Data were transferred from KoBoToolbox to Statistical Package for Social Sciences software (SPSS) version 26 for cleaning and analysis. Descriptive statistics were used to describe the study sample characteristics and mean for continuous variables. Categorical variables were compared using the Chi square test. Bivariate and multivariate statistics were conducted to assess for factors associated with adequate knowledge of CVDs risks factors. Variables with p ≤ .2 in the bivariate statistics were subjected to multiple logistic regression analysis for inferential analysis to determine the factors that were independently associated with adequate knowledge.

The Hosmer-Lemeshow test was employed to check the model’s fit in order to examine the accuracy of the logistic regression model. The Chi-square had 6 degrees of freedom, a value of 9.917, and a probability of P = .0.1491 which is greater than 0.05 in the test findings, showing that the logistic regression model was fit for the data set. The model explained 0.005 (Cox & Snell R Square) to 0.015 (Nagelkerke R Square) of the variation in the dependent variable, indicating that the logistic model explains 5–15% of the variation between the adequate and inadequate knowledge.

Odds ratios with their corresponding 95% confidence intervals and p-value were reported as measures of association. Statistical significance was accepted at p-value ≤ 0.05 on two-sided test.

## Results

### Characteristics of study participants

A total of 749 participants were enrolled in this study between March and May 2021. The response rate of 94%. Demographic characteristics of the study participants are described in Table 1. The age of respondents ranged from 19 to 72 years (mean ± standard deviation = 47.6 ± 14.3 years). More than half of respondents were female (57.9%). Nearly three quarters (71.7%) were married, moreover half (59.3%) had a primary education and majority (65.4%) were self-employed in small to large businesses, casual labor and farming activities.

**Table 1:** Demographic characteristics of the study participant (N = 749)

Characteristic	Proportion	%
Age in years [Mean (SD)]	47.6 ± 14.3	
**Age group (years)**		
19–24(youth)	16	2.1
25–44(young)	323	43.1
45–60(middle)	257	34.3
> 60(elderly)	153	20.4
**Sex**		
Female	434	57.9
Male	315	42.1
**Marital status**		
Single	103	13.8
Married	537	71.7
Divorced	50	6.7
Widowed	59	7.9
**Education level**		
No Formal	70	9.3
Primary	444	59.3
Secondary	138	18.4
College/University	97	13.0
**Occupation**		
Retired	89	11.9
Students	49	6.5
Self employed	490	65.4
Employed	121	16.2

Table 2 shows the prevalence of modifiable CVDs risk factors among study participants. Only 4.4% and 18.0% of the study participants were current smokers and alcohol drinkers respectively. Less than half (43.7%) of study participants reported to use raw table salt sometimes while about 20% reported to use it always. More than half of participants (56.9%) reported consuming fruit/vegetables less than 5 days per week. Over two-thirds (63.5%) of participants were in the category of overweight and obesity while 4.5% where underweight, 4.5% were diabetic and 43.4% were hypertensive. About 19% and 11% reported having a history of hypertension and diabetes in their family respectively.

**Table 2:** Prevalence of modifiable CVDs risk factors among study participants (N = 749)

Variables	Proportion (n)	%	
Behavioral risk factors			
Current smoking status			
Yes	33	4.4	
No	716	95.4	
**Current alcohol drinking status**			
Yes	135	18.0	
No	614	82.0	
**Use of raw table salt**			
Never	276	36.8	
Sometimes	327	43.7	
Always	146	19.5	
**Vegetable/fruit consumption**			
≥ 5 days/week	323	43.1	
< 5 days/week	426	56.9	
**Biological risk factors**			
**Body Mass Index [BMI (kg/m2]**			
Normal	239	31.9	
Overweight	252	33.6	
Obese	224	29.9	
Underweight	34	4.5	
**Diabetic**			
Yes	34	4.5	
No	715	95.5	
**Hypertensive**			
Yes	325	43.4	
No	424	56.6	
**Family history of hypertension**			
Yes	141	18.8	
No	608	81.2	
**Family history of Diabetes**			
Yes	85	11.3	
No	664	88.7	

### Knowledge of modifiable CVDs risk factors

Table 3 summarizes the results of participant’s responses to the 11 questions used to assess knowledge about CVDs risk factors. More than half (59.1%) of participants were aware that physical exercise can lower the CVDs risk. Smoking and second-hand smoke was known by 66.6% of study participants as a CVDs risk. Excessive alcohol intake was identified by 70.9% as a CVDs risk. More than half (58.6%) of participants were not aware that, regular consumption of red meat and sugary food is a CVDs risk. About 50% and 57% knew that, exercising regularly and eating a high fiber diet respectively is not a risk for CVDs risk. Consumption of too much salt was recognized by (62.9%) as a CVDs risk. About 63% were aware that walking and gardening are types of exercise that can lower CVDs risk and about 60% knew that consuming a lot of vegetables and fruits prevents the risk of CVDs. The lowest knowledge score was 0% and this was scored by 13 participants, which indicate that, 2.9% of study participants knew nothing about CVDs risk factors that were asked. The highest score was 100% which indicate that only 1.7% of the study participants had excellent understanding of CVDs risk factors. The mean knowledge score was 42.6%. The overall adequate knowledge score of risk factors for CVDs was 35.4%.

**Table 3:** Responses of the CVDs risk factors knowledge questionnaire used in this study (N = 749)

Item	Question	Correct answer	Answered incorrectly n (%)	Answered correctly n (%)
Qn01	Physical exercise can lower CVDs risk.	yes	443 (59.1)	306 (40.9)
Qn02	Smoking and second-hand smoke increases CVDs risk	yes	499 (66.6)	250 (33.4)
Qn03	Excessive alcohol intake increases CVDs risk	yes	531 (70.9)	218 (29.1)
Qn04	Regular consumption of red meat and sugary food is better for one’s healthy	no	310 (41.4)	439 (58.6)
Qn05	Eating a high fiber diet (e.g. brown bread, beans, un polished cereals) increases the risk CVDs	no	427 (57.0)	322 (43.0)
Qn06	Exercising regularly is harmful to cardiovascular health	no	377 (50.3)	372 (49.7)
Qn07	Consumption of too much salt is a risk to CVDs	yes	471 (62.9)	278 (37.1)
Qn08	Consuming a lot of vegetables and fruits prevents the risk of CVDs	yes	447 (59.7)	302 (40.3)
Qn09	Having excess body weight decreases one’s risk of CVDs	no	391 (52.2)	358 (47.8)
Qn10	Walking and gardening are types of exercise that can lower CVDs risk	yes	469 (62.9)	280 (37.4)
Qn11	Doing health check-ups frequently is harmful to cardiovascular health	no	361 (48.2)	388 (51.8)

Mean score ± SD = 42.6%±24.8%, minimum score = 0.0%, maximum score = 100%

### Factors associated with adequate knowledge of modifiable CVDs risk factors

Table 4 shows the results of chi-square analysis of various characteristics by adequate knowledge of CVDs risk factors status. Participants aged above 60 years had a higher likelihood (76.5%, p = .001) of having inadequate knowledge of CVDs risk factors compared to participants in other age groups. Male participants displayed higher rates of adequate knowledge of CVDs risk factors compared to female participants (40.3% vs. 31.8%, p = .016). Regarding marital status, participants who were widowed showed a higher chance (78.0%, p = .007) of having inadequate knowledge of CVDs risk factors compared to other participants in this category. Participants with primary education had a higher likelihood (68.5%, p < .001) of having inadequate knowledge of CVDs risks factors compared to other participants in this education level category. There was no much differences in adequate knowledge among the retired, students and self-employed participants except for the employed who displayed higher rates (51.2%, p = .001) of adequate knowledge of CVDs risk factors compared to the above-mentioned groups. Participants who had never checked their blood pressure had a higher chance (71.2% vs. 60.2%, p = .002) of having inadequate knowledge of CVDs risk factors compared to those who had checked their blood pressure. Likewise, Participants who had never checked their blood glucose showed a higher chance (67.8% vs. 58.9%, p = .014) of having inadequate knowledge of CVDs risk factors than those who had. Participants with a positive family history of hypertension displayed a higher rate (57.4% vs. 30.3%, p < .001) of adequate knowledge of CVDs risk factors compared to those who had no family history of hypertension. Similarly, participants with a positive family history of diabetes displayed a higher rate (50.6% vs. 33.4%, p = .002) of adequate knowledge of CVDs risk factors compared to those who had no family history of diabetes.

**Table 4:** Bivariate analyses of factors associated with adequate knowledge of CVDs risk factors among the vulnerable population in central Tanzania (N = 749)

Variable	Adequate knowledge 265 (35.4%)	Inadequate knowledge 484 (64.6%)	P-value
Age group (years)			
19–24(youth)	10 (62.5)	6 (37.5)	
25–44(young)	123(38.1)	200 (61.9)	
45–60(middle)	96 (37.4)	161 (62.6)	
> 60(elderly)	36 (23.5)	117 (76.5)	0.001
**Sex**			
Female	138 (31.8)	296 (68.2)	
Male	127 (40.3)	188 (59.7)	0.016
**Marital status**			
Single	48 (46.6)	55 (53.4)	
Married	191 (35.6)	346 (64.4)	
Divorced	13 (26.0)	37 (74.0)	
Widowed	13 (22.0)	46 (78.0)	0.007
**Education level**			
No Formal	6 (8.6)	64 (91.4)	
Primary	140 (31.5)	304 (68.5)	
Secondary	67 (48.6)	71 (51.4)	
College/University	52 (53.6)	45 (46.4)	< 0.001
**Occupation**			
Retired	28 (31.5)	61 (68.5)	
Students	15 (30.6)	34 (69.4)	
Self employed	160 (32.7)	330 (67.3)	
Employed	62 (51.2)	59 (48.8)	0.001
**Current smoking**			
Yes	20 (30.3)	23 (69.7)	
No	255 (35.6)	461 (64.4)	0.533
**Current alcohol drinking**			
Yes	56 (41.5)	79 (58.5)	
No	209 (34.0)	405 (66.0)	0.063
**Use of raw table salt**			
Never	99 (35.9)	177 (64.1)	
Sometimes	120 (36.7)	207 (63.3)	
Always	46 (31.5)	100 (68.5)	0.539
**Ever checked blood pressure**			
Yes	178 (39.8)	269 (60.2)	
No	87 (28.8)	215 (71.2)	0.002
**Ever checked blood glucose**			
Yes	111 (41.1)	159 (58.9)	
No	154 (32.2)	325 (67.8)	0.014
**Body Mass Index [BMI (kg/m2)]**			
Normal	84 (35.1)	155 (64.9)	
Overweight	95 (37.7)	157 (62.3)	
Obese	74 (33.0)	150 (67.0)	
Underweight	12 (35.3)	22 (64.7)	0.768
**Diabetic**			
Yes	9 (26.5)	25 (73.5)	
No	256 (35.8)	459 (64.2)	0.266
**Hypertensive**			
Yes	98 (30.2)	227 (69.8)	
No	167 (39.4)	257 (60.6)	0.009
**Family history of hypertension**			
Yes	81 (57.4)	60 (42.6)	
No	184 (30.3)	424 (69.7)	< 0.001
**Family history of Diabetes**			
Yes	43 (50.6)	42 (49.4)	
No	222 (33.4)	442 (66.6)	0.002

The results of logistic regression analysis are presented in Table 5. Twelve variables were entered into logistic regression model for analysis. Ten variables showed significant association with adequate knowledge of CVDs risk factors. After adjusting for the confounder variables (current alcohol drinking and diabetes) in the multivariate regression model, only six variables (age, sex, education level, occupation, ever checked BP and family history of high BP) remained independently associated with adequate knowledge of CVDs risk factors. When compared to their younger and elderly counterparts, the youth participants knew more about CVD risk factors. Men were more likely to have adequate knowledge of CVDs risk factors compared to women (AOR = 1.44, 95%CI = 1.01–2.06, p < .05). Participants who had primary education were 6 times more likely to have adequate knowledge of CVDs risk factors compared to participants who had no formal education (AOR = 6.43, 95%CI = 2.39–17.36, p < .0001). The employed participants had higher knowledge of CVDs risk factors compared to their counterpart the retired one (AOR = 1.59, 95%CI = 1.00-2.52, p < .05). Participants who had ever checked their blood pressure had higher odds of having adequate knowledge of CVDs risk factors than participants who had not ever checked their blood pressure (AOR = 0.59, 95%CI = 0.42–0.84, p < .001). Likewise, participants who reported to have family history of hypertension had higher odds of having adequate knowledge of CVDs risk factors compared to their counterparts who had not reported family history of hypertension (AOR = 0.38, 95%CI = 0.25–0.57, p < .0001).

**Table 5:** Multiple logistic regression model for predictors of adequate knowledge of CVDs risk factors among the vulnerable population in central Tanzania (N = 749)

Variable	OR	95%CI for OR		AOR	95%CI for AOR	
		Lower	Upper		Lower	Upper
Age group (years)						
19–24(youth)	ref			ref		
25–44(young)	0.19	0.06	0.54**	0.27	0.80	0.93*
45–60(middle)	0.50	0.32	0.77**	0.69	0.40	1.19
> 60(elderly)	0.51	0.33	0.81**	0.57	0.34	0. 94*
**Sex**						
Female	ref			ref		
Male	1.45	1.07	1.96*	1.44	1.01	2.06*
**Marital status**						
Single	ref			ref		
Married	0.32	0.16	0.67**	0.52	0.23	1.20
Divorced	0.51	0.27	0.92*	0.77	0.36	1.56
Widowed	0.80	0.33	1.94	1.08	0.42	2.79
**Education level**						
No Formal	ref			ref		
Primary	12.33	4.88	31.15***	6.43	2.39	17.34***
Secondary	2.51	1.61	3.92***	1.51	0.90	2.53
College/University	1.23	0.73	2,06	0.91	0.51	1.61
**Occupation**						
Retired	ref					
Students	2.29	1.29	4.06**	1.45	0.76	2.79
Self employed	2.38	1.18	4.82*	2.15	0.97	4.71
Employed	2.17	1.45	3.24***	1.59	1.00	2.52*
**Current alcohol drinking**						
Yes	ref					
No	0.73	0.49	1.07			
**Ever checked blood pressure**						
Yes	ref			ref		
No	0.612	0.45	0.84**	0.59	0.42	0.84**
**Ever checked blood glucose**						
Yes	ref					
No	0.679	0.50	0.93*	0.78	0.53	1.143
**Diabetic**						
Yes	ref					
No	1.55	0.71	3.37			
**Hypertensive**						
Yes	ref					
No	1.51	1.11	2.05**	1.32	0.93	1.88
**Family history of hypertension**						
Yes	ref					
No	0.321	0.22	0.47***	0.38	0.25	0.57***
**Family history of Diabetes**						
Yes	ref					
No	0.491	0.311	0.77**	0.70	0.42	1.18

Abbreviations: ref = reference, OR = Odds Ratio; AOR = Adjusted Odds Ratio; CI = Confidence Interval; *statistically significant association at *p-value < 0.05, **p-value < 0.001, ***p-value < 0.0001

## Discussion

The current study designed to assess the prevalence and knowledge of modifiable CVDs risk factors among the exposed population living in urban city of Dodoma, Tanzania’s central region. Overweight and obese, hypertension and low vegetable/fruit consuming were the most prevalent modifiable CVDs risk factors in this study. Overweight and obesity accounted for 63.5% of the total, with 33.6% being overweight and 29.9% being obese. These findings are comparable to those of Ajayi et al.’s study done in four sub-Saharan African adult populations of Nigeria, South Africa, Tanzania, and Uganda, which found that 31% of the adults were overweight and 34% were obese [24]. The prevalence of overweight was also comparable to that of Muhihi et al.’s study in Morogoro Tanzania, where the figure was 28.5% [10]. The overall prevalence of overweight and obesity was comparable to that of Kagaruki et al.’s study in Dar es salaam Tanzania, which reported the prevalence of 63.9% [25]. A high prevalence (75.9%) of overweight and obesity was also reported in the study of Iqbal Fahs et al. among the Lebanese Population [26]. The higher prevalence seen in this study is probably related to the consumption of unhealth diets combined with sedentary lifestyles and limited physical activity. This could be supported by the fact that in urban setting residence are busy for their earnings, spent much of their day on economic activities and having little time to prepare food of their own choice or preferences so they opt for ready-made food from food selling points. However, this study did not assess dietary quality and pattern, but Kagaruki et al., observed that, the quality of food sold by food selling points and street food vendors may serve as a facilitating or impeding environment for body weight gain or control [25]. Therefore, the study to assess the household dietary pattern among this community is recommended so as to establish the causal relationship.

The prevalence of hypertension (43.4%) in this study was comparable to that of Kagaruki et al. conducted in Dar es Salaam Tanzania, which reported the prevalence of 42.9% [25], but lower than other studies conducted in Tanzania [10, 15, 27]. The higher prevalence in this study may be explained by the rapid urbanization of Dodoma city which lead to changes of lifestyle such as eating habits to cope with the modern diets, this can also be reflected by the alarming rate of overweight and obesity in this population. Another possible explanation for the higher prevalence of hypertension in this study could be that, more than half (54.7%) of the study sample were the middle and older age population, female participants were more (57.9%) than men and the fact that females in this age group are past menopause this could have influenced the current prevalence. According to an epidemiological study, men have a higher prevalence of hypertension than women during their young adulthood, while women’s prevalence increases significantly after menopause [28].

This study documented low (43.1%) intake of fruit/vegetables at least 5 days per week among the study participants. This finding could probably due to insufficient income which may limit purchasing power and food choice in the family. On the other hand, the low intake of fruits and vegetables in this study could imply inadequate knowledge of study participants on the importance of fruits and vegetables in disease prevention and maintaining optimal health. Health program that will include the component of health education on healthy eating behavior will benefit this population. Studies have shown that, the consumption of recommended amount of fruits and vegetables reduces the incidence of type 2 diabetes, risk of cancer diseases and prevent weight gain [29–31]. The result of this study is similar to what has previously been reported in other part of the country [25, 32]. The result also is similar to the study done in South Africa by the South African National Health and Nutrition Survey 2013 which reported low intake of fruits and vegetables among study participants [33]. The World Health Organization reported that, intake of at least five portions of fruits and vegetables per day reduces the risk of non-communicable diseases and ensures sufficiency daily intake of dietary fiber [34]. Therefore, the study to assess cardioprotective servings ≥ 5 of fruits and vegetables per day in this study population is recommended so as to understand their portions intake and advice accordingly. The prevalence of diabetes in this study was higher than that of Tanzania national average [35]. However, it is consistent with other studies of urban site in African populations [36, 37]. This variations in the prevalence could be due to change in eating habits and sedentary lifestyle originating from increasing urbanization and economic development. Therefore, health promotion interventions at community level focusing on lifestyle modification could help to reduce the prevalence of the disease.

Only 35.4% of participants had adequate knowledge of modifiable CVDs risk factors while 2.9% of study participants were not able to identify any risk factor. Similarly, the study by Muhihi et al. [10]. found that 6.9% of study participants were unable to identify any risk factor. The low knowledge of CVDs risk factors in this study is comparable to other community-based studies conducted in African countries [38, 39]. The finding of this study is contrary to the study in Dar es Salaam Tanzania which found that 79.7% of study participants had good knowledge of CVDs risk factors [15]. The variations of the study finding in these two studies could be explained by the difference in study settings and the method used to assess knowledge. The study in Dar es salaam was a hospital-based study involved caretakers of patients attending a tertiary cardiovascular center hospital while the current study was a community-based study involved participants from the household. This could imply that, caretakers of patient attending cardiovascular hospital center could have acquired the knowledge through health education provided to their patients during clinic visits. Studies have reported that, health care providers as sources of information on CVDs risk factors [10, 40], unfortunately, individuals who receives knowledge of CVDs risk factors at the health facilities are the one who had already developed or have the risk factors. Therefore, interventions to focus on primary prevention at community level may increase population knowledge which could result in behavior change towards healthy lifestyle and consequently reduce the rising burden of CVDs in Tanzania. In the current situation whereby, the healthcare system in Tanzania is facing the burden of lack of human and financial resources for health, the suggested intervention could serve as a solution to reduce the burden of patients attending to the health facilities looking for CVDs services. Based on methods, the Dar es Salaam study tested risk factor knowledge across five domains (diet, epidemiology, risk factors, medical, and symptoms), whereas the current study assessed knowledge across three domains (diet, risk factors, and medical). This could have influenced the disparity in knowledge levels observed in these two researches. This study suggests doing another research in this cohort utilizing the five domains to evaluate whether there is a substantial change in knowledge level. This study found that the socio-demographic and biological characteristics of study participants were associated with knowledge of CVDs risk factors. The older and younger age participants were less knowledgeable on CVDs risk factors compared to the youth participants. Cognitive decline, which is frequent in the elderly, could explain this. Even after controlling for age and educational level, cognitive impairment can decrease performance across a wide range of cognitive domains, including attention, learning, and memory [41]. Prior research has indicated that decreases in executive function and memory might hamper clinical communication and compliance, as well as clinical decision-making [42], which could have contributed to the elderly’s lack of knowledge on CVDs risk factors in this study. People become more vulnerable to CVDs risk factors as they age [43], as does cognitive impairment [41]. CVDs are thought to hasten the deterioration of cognitive function, and impairment of cognitive domains raises the incidence of CVDs or worsens their prognosis [44]. This means that older people with less understanding of CVDs risk factors are more susceptible to CVDs, therefore, enhancing their CVDs knowledge may help to reduce their vulnerability to CVDs [45]. On the other hand, the young population < 45yrs mostly are still at low risk of CVDs hence less exposed to knowledge on the same compared to the middle aged adult who are at increased risk and some even already developed CVD conditions hence likely to be informed on the CVDs risks.

The finding of higher knowledge of CVDs risk factors among men is similar to the study done in Morogoro, Tanzania [10]. This is mostly due to men having more education and better wealth, which may lead to easier healthcare access where they can get information related to CVDs risk factors. Another possible explanation could be that men have ample time to listen, watch and read from mass media than women. One study found that more men than women stated that, radio, television and newspapers were their main sources of information on CVDs [10]. Contrary to this study, the study conducted in Kuwait found higher knowledge of CVDs risk factors among women than men [46]. This could be due to the high rate of CVDs literacy in their country. Therefore, community literacy on CVDs prevention is highly recommended. In this study the educated and the employed participants had adequate knowledge of CVDs risk factors. The findings of this study coincide to the findings of other studies conducted elsewhere [46–48]. This could imply that education and employment could help individuals to seek medical care and understand health information presented through various media. This study has found that participants who had ever checked their blood pressure and those who had a family history of hypertension had adequate knowledge on CVDs risk factors. This finding is congruent with other studies [10, 15]. This could suggest that, susceptibility to a health problem may lead an individual to seek for health information which may result in acquiring knowledge of a specific risk behaviors.

One of the study’s strengths is that all of the data gathering methods have been validated, translated, pre-tested, and adjusted to fit the study context. The knowledge tool was designed to be easily administered with true/false questions, which is essential when using actual scales to measure knowledge rather than self-reports. This scale has the potential to be a significant tool for both scholars and other practitioners. The tool comprised items with a wide variety of difficulties, resulting in the tool having maximal discrimination power throughout the spectrum of knowledge levels. This was beneficial in identifying knowledge gaps among research participants so that appropriate authorities could adapt health information. The tool is more comprehensive in that it measures knowledge of risk factors and CVDs prevention and it can be used to the majority of adult populations.

One of the weaknesses of the knowledge tool employed in this study is that the authors did not collect all five areas of the original tool (diet, epidemiology, risk factors, medical, and CVD symptoms). The research proposes another study in the same community that will use all five domains to assess if there is a significant difference in knowledge level between the current study and the planned study. Another weakness of this study is that, it did not assess the individuals’ dietary quality and eating habits, physical activity and economic situation which could function as an enabler for the prevalence of CVDs risk factors. Furthermore, the study did not assess the source of knowledge on CVDs risk factors, which could help in identifying the major sources of information for the community and developing novel measures to strengthen them. Despite these limitations the study provides the baseline estimate of the prevalence and understanding of modifiable CVDs risk factors in a large adult population of Dodoma City, where intervention to lower CVDs risk factors possible.

## Conclusion

In conclusion, the study has revealed high prevalence of biological (hypertension, diabetes, overweight and obesity) compared to behavioral (alcohol consumption, smoking and use of raw table salt) CVDs risk factors. The overall knowledge of modifiable CVDs risk factors in this study was inadequate. There seems to be a connection between inadequate knowledge of CVDs risk factors and high prevalence of modifiable CVDs risk factors in this study population. This may indicate lack of awareness and individual vulnerability to CVDs. Thus, innovative community educational strategies are desirable to create risk awareness in the community. These strategies will increase self-efficacy among individuals which may result to lifestyle modification and consequently reduce the risk of developing CVDs.

## Data Availability

The datasets used and/or analysed during the current study are available from the corresponding author on reasonable request.

## List of abbreviations

CVDs: Cardiovascular Diseases
RBG: Random Blood Glucose
FBG: Fasting Glucose Blood
BMI: Body Mass index
AOR: Adjusted Odds Ratio
OR: Odds Ratio
CI: Confidence Interval
